# Prospective Validation of Immunological Infiltrate for Prediction of Response to Neoadjuvant Chemotherapy in HER2-Negative Breast Cancer – A Substudy of the Neoadjuvant GeparQuinto Trial

**DOI:** 10.1371/journal.pone.0079775

**Published:** 2013-12-02

**Authors:** Yasmin Issa-Nummer, Silvia Darb-Esfahani, Sibylle Loibl, Georg Kunz, Valentina Nekljudova, Iris Schrader, Bruno Valentin Sinn, Hans-Ullrich Ulmer, Ralf Kronenwett, Marianne Just, Thorsten Kühn, Kurt Diebold, Michael Untch, Frank Holms, Jens-Uwe Blohmer, Jörg-Olaf Habeck, Manfred Dietel, Friedrich Overkamp, Petra Krabisch, Gunter von Minckwitz, Carsten Denkert

**Affiliations:** 1 German Breast Group, Neu-Isenburg, Germany; 2 Institute of Pathology, Charité-Universitätsmedizin Berlin, Berlin, Germany; 3 Department of Gynecology and Obstetrics, St. Johannes Hospital, Dortmund, Germany; 4 Frauenklinik Henriettenstiftung, Hannover, Germany; 5 Frauenklinik, Städtisches Klinikum Karlsruhe, Karlsruhe, Germany; 6 Sividon Diagnostics GmbH, Cologne, Germany; 7 Schwerpunktpraxis Bielefeld, Bielefeld, Germany; 8 Klinik für Frauenheilkunde und Geburtshilfe, Klinikum Esslingen, Esslingen, Germany; 9 Gemeinschaftspraxis für Pathologie Hamm, Hamm, Germany; 10 Breast Center, Helios-Klinikum Berlin-Buch, Berlin, Germany; 11 Gynäkologie und Geburtshilfe, St. Barbara-Klinik Hamm-Heessen, Germany; 12 Breast Center, St. Gertrauden Krankenhaus Berlin, Berlin, Germany; 13 Zentrum für Histopathologie Chemnitz, Chemnitz, Germany; 14 Oncologianova GmbH Recklinghausen, Recklinghausen, Germany; 15 Breast Center, Klinikum Chemnitz, Chemnitz, Germany; 16 University Women's Hospital, Frankfurt, Germany; National University of Ireland Galway, Ireland

## Abstract

**Introduction:**

We have recently described an increased lymphocytic infiltration rate in breast carcinoma tissue is a significant response predictor for anthracycline/taxane-based neoadjuvant chemotherapy (NACT). The aim of this study was to prospectively validate the tumor-associated lymphocyte infiltrate as predictive marker for response to anthracycline/taxane-based NACT.

**Patients and Methods:**

The immunological infiltrate was prospectively evaluated in a total of 313 core biopsies from HER2 negative patients of the multicenter PREDICT study, a substudy of the neoadjuvant GeparQuinto study. Intratumoral lymphocytes (iTuLy), stromal lymphocytes (strLy) as well as lymphocyte-predominant breast cancer (LPBC) were evaluated by histopathological assessment. Pathological complete response (pCR) rates were analyzed and compared between the defined subgroups using the exact test of Fisher.

**Results:**

Patients with lymphocyte-predominant breast cancer (LPBC) had a significantly increased pCR rate of 36.6%, compared to non-LPBC patients (14.3%, p<0.001). LPBC and stromal lymphocytes were significantly independent predictors for pCR in multivariate analysis (LPBC: OR 2.7, p = 0.003, strLy: OR 1.2, p = 0.01). The amount of intratumoral lymphocytes was significantly predictive for pCR in univariate (OR 1.2, p = 0.01) but not in multivariate logistic regression analysis (OR 1.2, p = 0.11).

**Conclusion:**

Confirming previous investigations of our group, we have prospectively validated in an independent cohort that an increased immunological infiltrate in breast tumor tissue is predictive for response to anthracycline/taxane-based NACT. Patients with LPBC and increased stromal lymphocyte infiltration have significantly increased pCR rates. The lymphocytic infiltrate is a promising additional parameter for histopathological evaluation of breast cancer core biopsies.

## Introduction

Primary systemic therapy is the treatment of choice in locally advanced breast cancer. Besides the well-established adjuvant therapy regimens neoadjuvant chemotherapy (NACT) is increasingly used in patients with operable cancers [1], [2]. While NACT of early stages of breast cancer leads to high clinical response rates [3], [4], a pathological complete remission (pCR) is achieved in only one-fourth of the patients, with variable rates in different subtypes.

The adaptive immune system is thought to play an important role in suppressing the progression of malignant cancers [5]–[9]. The presence of infiltrating lymphocytes within the tumor tissue has been shown for numerous tumor entities and high lymphocyte infiltration rates correlated with improved outcome [10]–[13]. For breast cancer patients older than 40 years a high degree of infiltrating lymphocytes was correlated with increased survival [14]. In rapidly proliferating breast cancer tissues, a lymphocytic infiltrate demonstrated to be an independent predictive indicator for recurrence-free survival [15]. Furthermore, we and others have shown that a high lymphocyte infiltration is predictive for response to NACT in breast cancer patients [16]–[19].

Using core biopsies of untreated breast carcinomas for the analysis of predictive markers, NACT regimen can be used as in vivo chemotherapy-sensitivity test with pCR as indicator of beneficial outcome from chemotherapy [20]. In previous retrospective investigations we could demonstrate that an increased immunological infiltrate is predictive for response after anthracycline/taxane NACT. We showed that lymphocyte-predominant breast cancer (LPBC), defined as tumors with >60% lymphocyte infiltrate of either stromal (strLy) or intratumoral (iTuLy) lymphocytes had a significantly increased pCR rate after NACT [16]. Using pretherapeutic core biopsies of HER2 negative patients randomized for the PREDICT study, a substudy of the neoadjuvant GeparQuinto trial, we prospectively analysed the immunological infiltration rate as independent predictor for response to NACT.

## Methods

### Study Population

A total of 313 FFPE primary tumor core biopsies were evaluated in the prospective PREDICT study, a substudy of the GeparQuinto trial. The GeparQuinto trial (NCT 00567554) was a prospective, randomized, open label, multicentre phase III trial program exploring the integration of Bevacizumab, Everolimus (RAD001) and Lapatinib into current neoadjuvant chemotherapy regimes for primary breast cancer. Chemotherapy consisted of 4 cycles of epirubicine, cyclophosphamide followed by taxane. The PREDICT study was designed as a substudy of GeparQuinto for prospective validation of molecular biomarkers in HER2 negative tumors in the neoadjuvant setting. Only HER2-negative patients in setting 1 that did not receive Bevacizumab were included in the PREDICT study. 93 centers (of a total of 127 GeparQuinto centers) have participated in the Predict substudy and have provided tumor samples in parallel to the randomization. Everolimus was administered to the non-responders in a second randomization, at that time the lymphocyte analysis had already been performed. 37 patients investigated for lymphocyte parameters were randomized to the Everolimus arm of GeparQuinto. Written informed consent for use of biomaterials was obtained from all patients, ethic committee approval was obtained for all centres participating in the clinical study and from the institutional review board of the Charité hospital.

### Data analysis approach

All clinical data, including the immunohistochemical data on estrogen receptor, progesterone receptor and HER2 status were extracted from the clinical study databases and represent the local assessment. This was predefined in the prospective statistical analysis plan for the PREDICT study.

### Tumor samples and inclusion criteria

All samples were formalin-fixed, paraffin-embedded pretherapeutic core biopsies collected before randomization, with written informed consent. Samples were stored in the GBG tumor bank at the Institute of Pathology, Charité Hospital, Berlin, Germany.

The following inclusion criteria were used: 1) HER2 negative patients that were randomized to setting 1 of GeparQuinto and did not receive Bevacizumab, 2) available primary tumor sample for biomarker analysis, 3) available data on pathological complete response (pCR). pCR was defined as the absence of residual invasive tumor cells in breast and lymph nodes (ypT0/is, ypN0).

### Sample preparation and immunological infiltration evaluation

Tissue samples were fixed in neutrally buffered formalin. From each paraffin block, a 2 µm section was prepared and stained with haematoxylin and eosin (H&E). Only samples with a proportion of tumor tissue of at least 30% of the whole tissue area were included in the analysis. Stromal Lymphocytes, intratumoral lymphocytes as well as LPBC were defined as previously described in Appendix Table A1 (online only) of [16], with one slight difference: Tumors with ≥60% intratumoral or stromal lymphocytes were designated as LPBC, this definition was more practical for routine assessment.

Intratumoral lymphocytes (iTu-Ly) were defined as intraepithelial mononuclear cells within tumor cell nests or in direct contact with tumor cells. They were reported as the percentage of the tumor epithelial nests that contain infiltrating lymphocytes. Stromal lymphocytes (str-Ly) were defined as the percentage of the tumor stroma area that contains a lymphocytic infiltrate without direct contact to tumor cells. The lymphocyte-predominant breast cancer (LPBC) was defined as tumors with either intratumoral lymphocytes in ≥60% of tumor cell nests or lymphocytes in ≥60% of the stromal area.

In this study the TILs were evaluated by only one pathologist. The interobserver variability for this evaluation has been tested by our group in the previous study, with a kappa score of 0.61 [16].

### Statistical analysis

Statistical analysis was performed using SAS 9.2 under SAS Enterprise Guide 4.3 (SAS Institute Inc., Cary, NC, USA). pCR rates were reported in subgroups defined by binary parameters (LPBC and clinical) and compared between subgroups using exact test of Fisher. The probability of pCR as a function of immunological parameters was determined by univariate logistic regression analysis. The multivariate logistic regression was used to adjust analysis for the known clinical parameters having influence on pCR.

## Results

### Baseline clinical data

**Figure 1:**
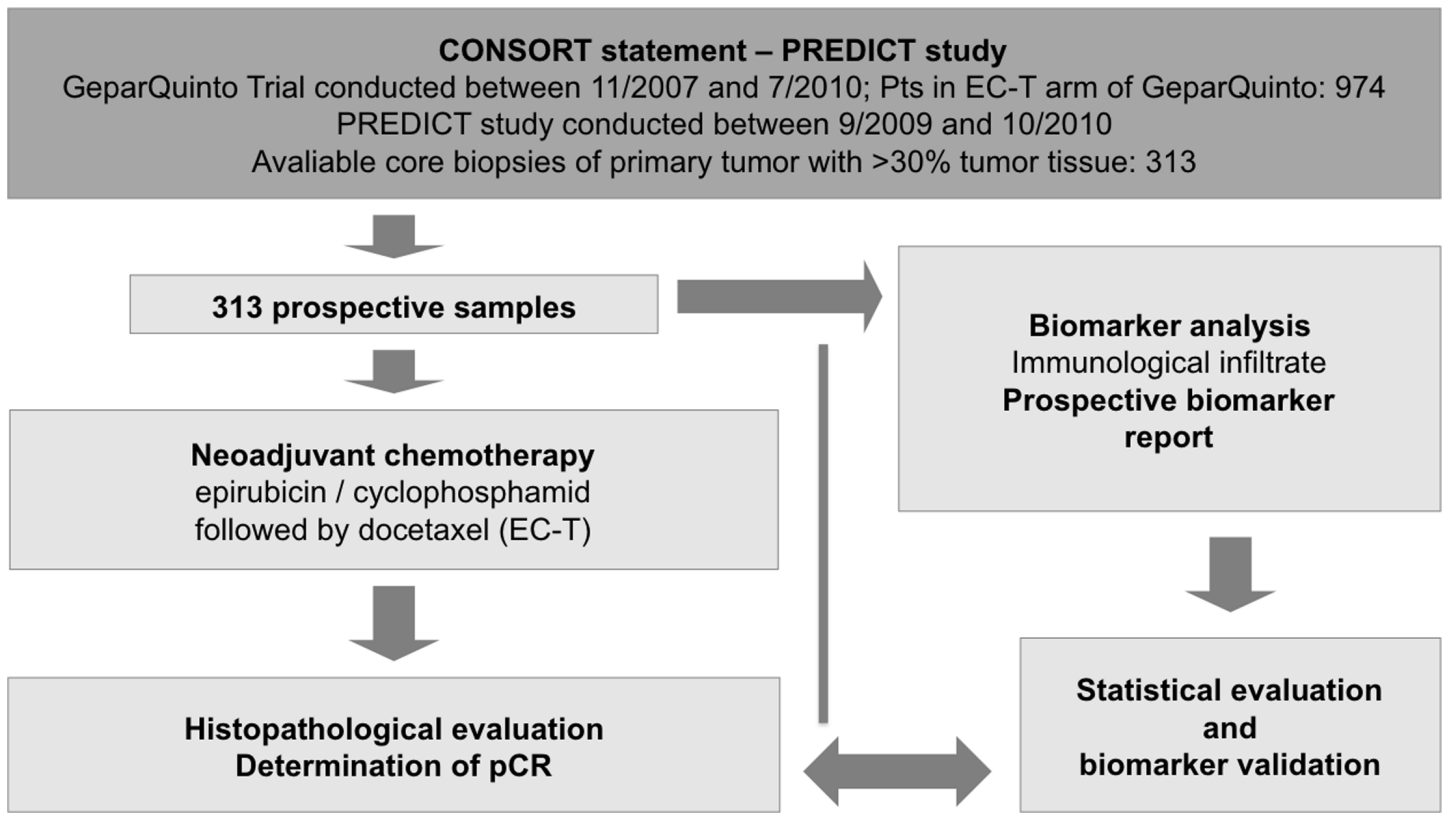
CONSORT statement and workflow of the PREDICT study. EC-T, epirubicin/cyclophosphamid followed by docetaxel; Pts, patients; pCR, pathologic complete response.

An independent prospective validation was performed in the PREDICT study, a substudy of the GeparQuinto trial. The CONSORT statement and workflow of the tumor samples are described in Figure 1. Between 9/2009 and 10/2010 a total of n = 313 HER2 negative patients were evaluated prospectively. Parallel to randomization, a FFPE tissue sample was sent to the central pathology laboratory by the site. The immunological infiltrate with the three parameters iTuLy, strLy and LPBC was evaluated in H&E sections. Histopathological results were sent as a report to the central trial office. The baseline characteristics of the patients are shown in Table 1. The rate of pCR, defined as the absence of residual invasive tumor cells in breast and lymph nodes, was 20.1% (63 of 313 patients, table 1). A total of 26.2% (82 of 313 patients) of all samples were classified as LPBC.

**Table 1:** Baseline characteristics of the PREDICT cohort.

Characteristic	PREDICT prospective validation cohort No. (%)
**No. of samples**	313
**Age group**	
<50 years	177 (56.5)
≥50 years	136 (43.5)
**Tumor type**	
ductal/other	278 (88.8)
lobular	34 (10.9)
missing	1 (0.3)
**Tumor grade**	
G1–G2	177 (56.5)
G3	136 (43.5)
**ER/PR** * ** Status**	
ER−/PR−	104 (33.2)
ER+ and/or PR+	209 (66.8)
**HER2 status**	
HER2 negative	313 (100)
HER2 positive	0 (0)
**Clinical tumor stage**	
cT1–cT2	267 (85.3)
cT3–cT4	46 (14.7)
**Clinical nodal status**	
cN0	132 (42.2)
cN+	170 (54.3)
missing	11 (3.5)
**LPBC status**	
negative	231 (73.8)
positive	82 (26.2)
**Pathological response**	
no pCR	250 (79.9)
pCR	63 (20.1)

* Abbreviations: ER: estrogen receptor; PR progesterone receptor:

LPBC: lymphocyte predominant breast cancer,

pCR: pathological complete response.

### Prospective evaluation of the immunological infiltrate in the GeparQuinto PREDICT substudy

High-grade tumors had a significantly increased pCR rate of 30.9% compared to the group of G1–G2 tumors with a pCR rate of 11.9% (G3 vs. G1–G2 30.9% vs. 11.9%, p<0.001). Hormone receptor negative tumors showed a significant increase of pCR rate (ER−/PR− vs. any+ 36.5% vs. 14.3%, p<0.001). The pCR rate of invasive ductal carcinomas was 21.9%, compared to 2.9% for invasive lobular tumors (p = 0.01). The pCR rate of nodal negative patients was significantly increased compared to patients with tumor infiltrated lymph nodes (cN0 vs. cN+ 26.5% vs. 15.3%, p = 0.02). The evaluation of tumor stage (cT1–2 vs. cT3–4) and age of the patients (<50 vs. ≥50 years) showed no significant influence on the pCR rates after NACT (Table 2).

**Table 2:** Evaluation of pathological complete response (pCR) to neoadjuvant chemotherapy.

PREDICT study cohort	n	pCR (%)	p-value*
**iTuLy (continuous)** per 10%	313	-	-
**strLy (continuous)** per 10%	313	-	-
**LPBC** (pos. vs. neg.)	313	36.6 vs. 14.3	<0.001
**HR status** (ER−/PR− vs. any+)	313	36.5 vs. 12.0	<0.001
**Age group** (<50 vs. ≥50 years)	313	22.0 vs. 17.6	0.4
**Tumor type** (ductal/other vs. lobular)	312**	21.9 vs. 2.9	0.01
**Tumor grade** (G3 vs. G1–G2)	313	30.9 vs. 11.9	<0.001
**Tumor stage** (cT1–2 vs. cT3–4)	313	21.7 vs. 10.9	0.11
**Clinical nodal status** (cN0 vs. cN+)	313	26.5 vs. 15.3	0.02

* p-values: Fisher's exact test, 2-sided.

** one tumor type was missing.

The group of lymphocyte-positive breast cancer (LPBC) had a significantly increased pCR rate of 36.6%, whereas non-LPBC tumors had a pCR rate of 14.3% (LPBC pos. vs. neg. 36.6% vs. 14.3%, p<0.001).

Table 3 shows the association between LPBC and clinical parameters, LPBC was significantly increased in the hormone receptor negative groups and in high grade tumors.

**Table 3:** Association of LPBC status with clinical parameters.

	LPBC negative (n, %)	LPBC positive (n, %)	p-value*
**HR status**			<0.001
negative (ER−/PR−)	66 (63.5)	38 (36.5)	
positive (any+)	184 (88.0)	25 (12.0)	
**Age group**			0.394
<50 years	138 (78.0)	39 (22.0)	
≥50 years	112 (82.4)	24 (17.6)	
**Tumor type**			0.006
ductal/other	217 (78.1)	61 (21.9)	
lobular	33 (97.1)	1 (2.9)	
**Tumor grade**			<0.001
G1–G2	156 (88.1)	21 (11.9)	
G3	94 (69.1)	42 (30.9)	
**Tumor stage**			0.111
cT1–2	209 (78.3)	58 (21.7)	
cT3–4	41 (89.1)	5 (10.9)	
**Clinical nodal status** (vs.			0.020
cN0	97 (73.5)	35 (26.5)	
cN+	144 (84.7)	26 (15.3)	

* p-value: two-sided Fisher test.

In logistic regression analysis (Table 4), all three immunological parameters (iTuLy, strLy and LPBC) were significant independent parameters for pCR in univariate analysis per 10% increase in lymphocytic infiltrate (iTuLy OR 1.2, 95% CI 1.1–1.5, p = 0.01; strLy OR 1.2, 95% CI 1.1–1.4, p<0.001; LPBC OR 3.5, 95% CI 1.9–6.2, p<0.001). Other independent parameters in univariate analysis were negative hormone receptor status (OR 4.2, 95% CI 2.4–7.6, p<0.001), tumor grade (G3 vs. G1–G2, OR 3.3, 95% CI 1.9–6.0, p<0.001) and negative nodal status (OR 2.0 CI 95% 1.1–3.5, p = 0.02).

**Table 4:** Univariate and multivariate analysis.

	Univariate analysis*		Multivariate analysis without lymphocyte parameters		Multivariate analysis incl. iTuLy		Multivariate analysis incl. strLy		Multivariate analysis incl. LPBC	
	OR (95% CI)	p-value	OR (95% CI)	p-value	OR (95% CI)	p-value	OR (95% CI)	p-value	OR (95% CI)	p-value
**iTuLy (continuous) per 10%**	1.2 (1.1–1.5)	0.01			1.2 (0.97–1.4)	0.11	-		-	
**strLy (continuous) per 10%**	1.2 (1.1–1.4)	<0.001			-		1.2 (1.0–1.3)	0.01	-	
**LPBC** (pos. vs. neg.)	3.5 (1.9–6.2)	<0.001			-		-		2.7 (1.4–5.2)	0.003
**HR status** (ER−/PR− vs. any+)	4.2 (2.4–7.6)	<0.001	2.52 (1.31, 4.86)	0.006	2.3 (1.2–4.5)	0.01	2.3 (1.2–4.5)	0.02	2.4 (1.2–4.6)	0.01
**Age group** (<50 vs. ^3^ 50 years)	1.3 (0.75–2.3)	0.34	1.21 (0.65, 2.26)	0.56	1.3 (0.67–2.4)	0.48	1.3 (0.66–2.4)	0.48	1.2 (0.65–2.4)	0.51
**Tumor type** (ductal/other vs. lobular)	9.3 (1.2–69.2)	0.03	5.66 (0.73, 43.8)	0.10	5.3 (0.69–41.4)	0.11	5.0 (0.65–39.1)	0.12	5.0 (0.64–39.0)	0.13
**Tumor grade** (G3 vs. G1–G2)	3.3 (1.9–6.0)	<0.001	2.02 (1.03, 3.96)	0.04	1.9 (1.0–3.7)	0.07	1.6 (0.79–3.2)	0.19	1.6 (0.78–3.2)	0.21
**Tumor stage** (cT1–2 vs. cT3–4)	2.3 (0.86–6.0)	0.10	1.79 (0.64, 5.06)	0.27	1.7 (0.58–4.7)	0.34	1.6 (0.55–4.5)	0.39	1.6 (0.55–4.5)	0.40
**Clinical nodal status** (cN0 vs. cN+)	2.0 (1.1–3.5)	0.02	1.73 (0.93, 3.22)	0.084	1.9 (1.0–3.5)	0.05	2.0 (1.0–3.7)	0.04	1.9 (1.0–3.5)	0.06

* p-value: logistic regression analysis.

The multivariate analysis revealed LPBC and increased stromal lymphocytes (strLy) as significant independent predictors for pCR with an OR of 2.7 (95% CI 1.4–5.2, p = 0.003) and OR of 1.2 (95% CI 1.0–1.3, p = 0.01), respectively. The presence of intratumoral lymphocytes (iTuLy) was no significant independent parameter for pCR in multivariate analysis (OR 1.2, 95% CI 0.97–1.4, p = 0.11). Another significant independent parameter in multivariate testing was a negative hormone receptor status (p<0.05, table 4).

In an exploratory analysis, we have analyzed the pCR rate according to hormone receptor status: In our cohort, HR positive tumors had a pCR rate of 12%, while HR negative tumors had a pCR rate of 36.5%. In HR positive tumors (n = 209), the pCR rate for LPBC was increased to 28.2% (11 pCRs of 39 tumors), while it was only 8.2% (14 pCRs of 170 tumors) for non-LPBC tumors (p = 0.002, Fisher test 2-sided). For the HR negative tumors (n = 104) the pCR rate was increased to 44.2.% for LPBC, compared to 31.1% for non-LPBC (p = 0.22, Fisher test).

## Discussion

Previous data from our group demonstrated that the degree of lymphocyte infiltration can be used as a continuous predictive factor for response to NACT. Tumors with a particular strong lymphocytic infiltrate, designated LPBC, had a significantly increased pCR rate compared to tumors with low lymphocyte infiltration [16]. In this study we could verify this finding in an independent, prospectively assessed cohort. LPBC and increased stromal lymphocytes were as significant independent predictors for pCR in multivariate analysis. However, the presence of iTuLy was significant for pCR only in univariate but not in multivariate logistic regression analysis. It should be noted that our investigation was performed on core biopsies and that evaluation of the immunological infiltrate could be different in large tumor sections. Furthermore, this infiltrate is a continuous parameter reflecting a biological characteristic. The designation LPBC just refers to the extreme variant of this continuous parameter. LPBC can therefore be used as a working category to facilitate diagnostic assessment. In our study the pCR rate was significantly increased in our cohort only in the HR positive subset. The non-significant increase in HR negative tumors could be due to the smaller size of this group and the already comparably high pCR rates in the non-LPBC tumors. As we did not perform a survival analysis, the prognostic effect of TILs cannot be evaluated in our study.

There are several distinct mechanisms of the tumor-immune interaction in response to chemotherapy that may be considered for our results. Chemotherapy can increase the susceptibility of tumor cells to lysis by cyctotoxic CD8^+^ T cells mediated by DNA-damaging agents such as cyclophosphamide [21]. Low doses of cyclophosphamide were demonstrated to selectively reduce circulating regulatory T cells (Treg cells) but restore T cell and natural killer (NK) cell functions [22]. Anthracycline-treated tumor cells are particularly effective in eliciting an anti-tumor immune response. Data from Ladoire et al. (2008) showed that a systemic anthracycline-based NACT in early-stage breast cancer patients resulted in a decreased infiltration of Treg cells whereas the level of CD8^+^ T cell infiltration remained unchanged in complete responders [23]. Furthermore, taxanes can stimulate the proliferation of T cells and the cytolytic activity of NK cells in adjuvant treated breast cancer patients [24]. In the GeparQuinto trial all patients had received an anthracycline/cyclophosphamide-based regimen followed by paclitaxel treatment. Our previous retrospective analysis investigated differently treated cohorts from the GeparDuo and GeparTrio trials that received three different major therapy regimens but still demonstrated similar immunological effects [16], [25], [26]. Taken together with our here demonstrated results this fosters the hypothesis that additionally to the directly induced stimulating effects of one or more chemotherapeutic reagents a subsequent immunological reaction may be induced by the destruction of tumor cells and release of specific tumor-associated antigens [27].

The large group of infiltrating lymphocytes can encompass T cells as well as B cells with their respective different subgroups. It is known that the composition of the immune infiltrate can influence the prognostic and/or predictive impact in different tumor types including breast carcinoma [28]–[31]. Several studies reported that cytotoxic CD8^+^ T cells and T helper CD4^+^ T cells infiltrated in the tumor tissue lead to a reduction of the tumor growth [32]. In contrast, CD4^+^CD25^high^FoxP3^+^ regulatory T cells (Treg cells) can stimulate tumor growth [33], [34]. Mahmoud et al. (2011) investigated the predictive impact of tumor-infiltrated cytotoxic CD8^+^ T cells on the clinical outcome in 1334 breast cancer patients. The authors demonstrated that the rate of tumor-infiltrating cytotoxic CD8^+^ T cells significantly correlates with improved clinical outcome [31]. Treg cells are thought to modulate the anti-tumor immune response by suppressing the activity of cytotoxic CD8^+^ T cells through direct cell-cell contact and/or secretion of transforming growth factor β (TGFβ). High infiltration rates of Treg cells were found in the peripheral blood as well as in the tumor tissue in a variety of tumors including breast cancer [35], [36]. A correlation of a high Treg cell infiltration in breast tumor tissue with worse outcome for the patients was demonstrated by different authors [37], [38]. Furthermore, Bates et al. (2006) showed that high numbers of tumor-infiltrating Treg cells is an independent prognostic factor for shorter recurrence-free survival and overall survival in patients with ER+ breast cancers [39].

Increased levels of mature B cells can be located in secondary lymphoid tissues as well as in the tumor tissue of different tumor entities including breast cancer [40]–[42]. Although the role of tumor-infiltrating B cells is still not clear, it is suggested that activated B cells may contribute to an anti-tumor immune response by secretion of antigen-specific antibodies, induction of innate immune cells (e.g. M1 tumor-associated macrophages), release of distinct cytokines (e.g. IL-6) and activation of complement cascades [13], [43]. Activated B cells can also function as antigen-presenting cells to induce tumor-specific cytotoxic T cells and therefore contribute to cellular immunity [43], [44]. Moreover, it is claimed that tumor-infiltrating B cells possibly activate humoral immunity within the tumor tissue by the induction of an antibody response against tumor-associated antigens [45]–[48]. Recently, Mahmoud et al. (2011) revealed in a large study of 1470 primary invasive breast cancer tissues that high numbers of tumor-infiltrating B cells correlated with a good prognosis for the patients [49]. Furthermore, an analysis of gene expression patterns of 200 node-negative breast tumor patients identified a positive association of tumor-infiltrating B cells with the survival of the patients [50].

In our current data we evaluated the lymphocytic tumor-infiltrate without further identification of the subgroups of the different T and B cell subpopulations. Nevertheless, our previous investigation analyzed immunohistochemically the composition of the lymphocytic infiltrate consisting of CD20 positive B cells and CD3 positive T cells [16]. As described, the different lymphocytic patterns of T and B cells subpopulations could play an important role in the tumor evasion strategy based on the immune status. It is implicated that besides the directly induced effects of chemotherapeutic reagents the cellular immune response as well as the adaptive humoral immune answer may have an additional effect in anti-tumor response in the LPBC and strLy subgroups. Therefore the impact on the response to NACT of breast cancer patients may be beneficial. To verify this hypothesis further investigation is needed. A further stratification of the different lymphocyte subpopulations might increase the accuracy for prediction to response to NACT.

In conclusion, we show in a prospectively evaluation that tumor-associated lymphocyte infiltration in breast carcinoma tissue is a predictor for response to NACT. Confirming our previous data, we demonstrated that an increased lymphocytic infiltration rate is predictive for response to anthracycline/taxane-based NACT. Our findings suggest that the comparably simple evaluation of tumor-associated lymphocytes in H&E sections could be used as an additional parameter to define a group of patients that might benefit from NACT.
